# Deciphering the Relevance of Bone ECM Signaling

**DOI:** 10.3390/cells9122630

**Published:** 2020-12-07

**Authors:** Natividad Alcorta-Sevillano, Iratxe Macías, Arantza Infante, Clara I. Rodríguez

**Affiliations:** Stem Cells and Cell Therapy Laboratory, Biocruces Bizkaia Health Research Institute, Cruces University Hospital, Plaza de Cruces S/N, Barakaldo, 48903 Bizkaia, Spain; natividad.alcortasevillano@osakidetza.eus (N.A.-S.); iratxe.maciasgarcia@osakidetza.eus (I.M.)

**Keywords:** ECM, bone fragility, fracture risk, bone disease, ECM signaling

## Abstract

Bone mineral density, a bone matrix parameter frequently used to predict fracture risk, is not the only one to affect bone fragility. Other factors, including the extracellular matrix (ECM) composition and microarchitecture, are of paramount relevance in this process. The bone ECM is a noncellular three-dimensional structure secreted by cells into the extracellular space, which comprises inorganic and organic compounds. The main inorganic components of the ECM are calcium-deficient apatite and trace elements, while the organic ECM consists of collagen type I and noncollagenous proteins. Bone ECM dynamically interacts with osteoblasts and osteoclasts to regulate the formation of new bone during regeneration. Thus, the composition and structure of inorganic and organic bone matrix may directly affect bone quality. Moreover, proteins that compose ECM, beyond their structural role have other crucial biological functions, thanks to their ability to bind multiple interacting partners like other ECM proteins, growth factors, signal receptors and adhesion molecules. Thus, ECM proteins provide a complex network of biochemical and physiological signals. Herein, we summarize different ECM factors that are essential to bone strength besides, discussing how these parameters are altered in pathological conditions related with bone fragility.

## 1. Introduction

The bone mineralized extracellular matrix (ECM) is predominantly responsible for bone’s resistance to fracture, defined as bone strength. Bone formation or internal reconstruction will determine not only the spatial structure of the tissue but its mechanical properties. Bone mass has been used as a predictor of bone fragility; however, it is only a partial correspondent. Indeed, the skeleton derives its resistance to fracture from multiple components regulated across several levels of hierarchical organization. That way, the relative composition, organization, and maturity of the mineral and organic matrix have a paramount relevance on how bones respond to mechanical demand.

### 1.1. Bone Extracellular Matrix Composition

Bones involve living cells embedded in a mineralized matrix, consisting of organic and inorganic phase [1]. While the inorganic matrix is responsible for the ability to resist deformation (bone strength and stiffness), organic matrix allows energy absorption (toughness) [2]. The cellular component of bone is in constant interaction with the surrounding ECM, which affects cellular function by regulating different signaling pathways. All in all, different cells and molecules that compose bone matrix are involved in bone strength and, therefore, alterations in either fraction may affect bone composition and mechanical properties, determining fracture risk [3].

#### 1.1.1. Inorganic Matrix

The inorganic (or mineral) fraction of bone tissue, composed of a combination of calcium and phosphorus salts, (predominantly in the form of hydroxyapatite (Ca_10_(PO_4_)_6_(OH)_2_)), is of ultimate importance to bone strength and stiffness. Crystals of calcium phosphate, produced by osteoblasts, are laid down in precise amounts within the fibrous matrix, leading to bone mineralization (also known as calcification). Mineral is initially deposited between the ends of collagen fibrils of the matrix, whilst during bone maturation hydroxyapatite crystals grow and aggregate [4].

When the maturation process occurs, expressed proteins regulate ordered deposition of mineral by regulating the amount and size of hydroxyapatite crystals formed. Two proteins appear essential in bone mineralization: type I collagen, which constitutes the scaffold upon which mineral is deposited, and alkaline phosphatase, that hydrolizes pyrophosphate (a strong inhibitor of mineralization) plus modifies the phosphorylation status of osteopontin (OPN), a factor implicated in bone remodeling [5]. Other bone matrix proteins are also known to regulate the mineralization process such as proteoglycans [6], matrix Gla-protein [7] and various phosphate-regulating proteins. Bone mineralization is also controlled by systemic hormones such as parathyroid hormone (PTH) and vitamin D [8]. PTH, the principal regulator of calcium homeostasis, enhances the release of calcium from the large reservoir contained in the bones [9] whilst, vitamin D stimulates the intestinal absorption of calcium and phosphorus to achieve enough calcium concentration [10]. Even more, the later also promotes differentiation of osteoblasts, stimulating the expression of bone crucial players; such as bone-specific alkaline phosphatase, osteocalcin (OC) and osteonectin (ON), among others.

Finally, the degree of mineralization, closely linked with bone strength [11], is mostly determined by the rate of bone turnover [12]. High bone turnover decreases the overall bone mineralization leading to lower bone stiffness. On the contrary, a reduced bone turnover leads to the accumulation of older and more extensively mineralized bone [12], with the consequent biomechanical drawbacks: it makes bone more brittle [13] and leads to the accumulation of damaged (aged) bone with reduced elastic properties, facilitating microcrack and fracture occurrence. Therefore, adequate balance between bone formation and resorption is crucial for bone quality [14].

#### 1.1.2. Organic Matrix

Proteins that compose bone ECM can be divided into collagen and, to a minor extent, other noncollagenous proteins (NCPs). Bone-forming cells (osteoblasts) secrete the main compound of the organic matrix: type I collagen, which constitutes about 85–90% of the total bone protein content. Type I collagen, encoded by *COL1A1* and *COL1A2* genes, not only plays a major structural role in bone but also contributes to tissue organization and therefore to its mechanical properties [15]. Type I collagen is first synthesized as the precursor procollagen, being subsequently stabilized by post-translational modifications and disulfide bonds. Then, it is secreted into the ECM, cleaved of the N- and C-terminals, and processed until native triple helix collagen is obtained.

NCPs, such as proteoglycans, SIBLING proteins (small integrin-binding ligand, N-linked glycoproteins), glycosylated proteins, γ-carboxylated proteins, and other serum-derived proteins, are present in the bone matrix taking part in collagen assembly and crosslink formation [16] affecting the mechanical properties of collagen. This way, abnormalities in collagen crosslinks have been associated with increased fracture risk [17,18]. 

All in all, the correct synthesis and fiber orientation of collagen are mandatory to obtain a healthy bone matrix able to withstand bone tensile strength. As such, it is not surprising that defects in type I collagen have dramatic effects on the skeleton. 

#### 1.1.3. Cellular Components

Bone is additionally composed of four different cell types that are in constant interaction with the surrounding ECM [19]. First, osteoprogenitor cells have the capacity to divide and differentiate into different bone cells. These cells, also known as mesenchymal stem cells (MSCs), differentiate to osteoblasts under osteogenic conditions. Osteoblasts are bone forming cells that synthesize and secrete the collagen matrix plus accomplish the mineralization of bone matrix. Then, when the secreted matrix surrounding the osteoblast calcifies, the osteoblast becomes trapped within it. As a result, it changes in morphology, becoming an osteocyte, the primary cell of mature bone that maintains the bone tissue. Finally, osteoclasts, multinucleated cells derived from hematopoietic progenitors, are the responsible for bone tissue degradation. Since bone is a dynamic tissue, bone remodeling is tightly regulated by both osteoblasts and osteoclasts: while osteoblasts form new bone, osteoclasts resorb it.

### 1.2. Bone Structure: Microarchitecture

Overall, the human skeleton is composed of bones grouped in four categories: long bones (femur, tibia, clavicles), short bones (for instance carpal and tarsal bones), flat bones (such as the ribs, mandible and skull) and irregular bones (such as vertebrae). All of them are composed of two types of bone tissue which can be distinguished macroscopically, differing in their architecture but similar in molecular composition: cortical (or compact) bone and trabecular (or cancellous) bone (80% and 20% of human skeleton, respectively) [20]. Although composed by the same components, mainly hydroxyapatite, collagen and water, trabecular bone is less mineralized (it has lower calcium content and higher water content), presenting lower tissue density and mineral content compared to cortical bone [21]. Consequently, cortical bone is densely packed, providing the strength and rigidity to bones. On the contrary, trabecular bone, responsible for the most bone turnover [22], is a porous material composed of a network of trabeculae organized to optimize load transfer, dispersing the energy of loading [23]. The cortical to trabecular ratio in each bone varies depending on the bone type and the specific skeletal site of that bone. Thus, cortical bone is mainly present in shafts of long bones and outer surfaces of flat bones, whereas trabecular bone is found at the end of long bones, vertebral bodies and the inner part of flat bones. 

Alterations in bone ECM components can disrupt ECM-bone cell signaling leading to deterioration of bone mineral density (BMD) (the content of calcium in a certain volume of bone) and/or bone microarchitecture, (the organization of bone components in space), the two main parameters determining bone strength. In vivo quantification of cortical and trabecular BMD, geometry and microarchitecture can be analyzed at the same time by quantitative computed tomography methods, rendering the amount of cortical and trabecular bone tissue and features of trabecular (trabecular number, trabecular thickness, trabecular separation) and cortical (cortical thickness and porosity) bone microarchitecture. 

### 1.3. Biophysical Properties of Bone Extracellular Matrix

A growing body of evidence in ECM biology points at biophysical properties of the bone ECM (mineral crystal size, their crystallinity (the degree of structural order) and the degree and type of collagen crosslinking,) as important determinants of cell behavior. Indeed, every cell in its anatomical localization has to balance the external forces dictated by the mechanical properties of its environment, which results from the compression exerted by neighboring cells as well as the stiffness of the surrounding ECM.

Regarding the biophysical properties of the mineralized matrix that surrounds bone cells, not only does the degree of mineralization matter so does the individual characteristics of the hydroxyapatite crystals (their size and shape) and crystallinity. Indeed, excessive crystal growth damages collagen fibers, affecting the tissue mechanical properties. Moreover, bone strength seems to be favored by greater mineral crystal size heterogeneity [24]. 

The biophysical properties of collagen type I fibers affect cellular behaviors [25], since cells respond differently to denatured collagen than to mature, crosslinked collagen fibrils [26]. Collagen crosslinking is a major post-translational modification which determines biophysical properties such as tensile strength and viscoelasticity [17]. Crosslinks can be divided into enzymatic and nonenzymatic. Enzymatic crosslinking is a process in which the ends of the collagen molecules are linked (so their number is greatly limited), acquiring a more stable, trivalent, nonreducible conformation [27]. When mature crosslinks accumulate, collagen fibril remodeling is inhibited and stiffness of the fibril increased, providing strength to the tissue [28]. That way, enzymatic crosslinking bears beneficial effect on the mechanical properties of collagen [17]. Conversely, nonenzymatic crosslinking does not involve any enzymes, and are found at any position along the collagen molecule to connect either collagen molecules or fibrils. Nonenzymatic glycation results in the formation of intermediate products (advanced glycation end-products (AGEs)) that undergo additional reactions to create crosslinks that form within and across collagen fibers. Thus, nonenzymatic crosslinking results in a brittle collagen network that, when accumulated or when its spatial distribution is altered leads to deteriorated bone mechanical properties [29,30]. In summary, while enzymatic crosslinking of collagen is generally considered to have a positive effect on bone´s mechanical properties, nonenzymatic crosslinking can lead to deteriorated bone mechanical properties.

### 1.4. Bone Extracellular Matrix Signaling

As previously mentioned, the majority of bone ECM is composed by collagen type I, reaching up to 90% of the protein content. However, proteomic analysis of decalcified bone has identified the minority presence of more than 100 ECM proteins in bone, different from collagen, reflecting the complexity of bone ECM [31,32]. 

In addition to structural role and thanks to their ability to bind multiple interacting partners like other ECM proteins, growth factors, signal receptors and adhesion molecules [33], the diverse set of ECM proteins also reveal other crucial biological functions. ECM components thus, provide a complex network of biochemical and physiological signals that contribute to bone metabolism, affecting fundamental cellular processes (such as proliferation, differentiation, migration and survival) via the integration of a number of signals that constitute the matrix-to-cell signaling [33]. This way, ECM regulates both, the osteoblast-lineage (for instance progenitors, mature osteoblasts, and osteocytes) and osteoclast-lineage (including precursors and mature osteoclasts), including also the crosstalk between them [34]. Besides, external influences can exert changes in these complex signaling systems as for instance vitamins [35] hormones [36,37] and/or minerals [38] intake.

In this section, we will highlight the main pathways that are involved in bone ECM signaling to offer a better understanding of how cell-matrix signaling occurs and the relevance of thereof in pivotal biological processes. 

#### 1.4.1. Integrin-Dependent Cell Adhesion Structures in Cell-ECM Signaling

Cell migration, essential for embryonic development, tissue renewal and immune response among other key processes, becomes crucial for correct bone remodeling. The formation of new bone needs the migration and differentiation of MSCs, an event tightly controlled by sequential activation of diverse transcription factors which regulates the expression of specific genes responsible for this transition [39]. The activation of these signaling cascades, and thus cell fate, is governed by the integration of all the signals that the cell receives from its environment through the ECM and intercellular adhesions.

Integrin-dependent cell adhesion structures allow cells to be attached to the ECM, binding intracellular actin fibers to extracellular proteins like fibronectin. This connection also transmits the mechanical force and regulatory signals between the ECM and the cytoskeleton of the cells. 

Integrins are heterodimeric transmembrane receptors formed by one α and one β subunit. There are several subunit isoforms (eighteen α and eight β) that can be noncovalently assembled into 24 combinations [33] and the exact subunit combination dictates their binding specificity to different ECM components. Within the cell, the intracellular domain do not bind directly to the cytoskeleton, they do so via adapter proteins such as talin, α-actinin, filamin, vinculin and tensin [40,41], which transmit the applied forces on ECM to the actin cytoskeleton. Conversely, forces applied to actin, the so-called ‘traction forces’, are also transmitted to the ECM through the same mechanism [42]. 

As mentioned, integrins can be assembled into several combinations that are different in their mechanosensitivity and elicited cellular responses. Mechanosensation depends on ECM material properties, being broadly demonstrated that ECM stiffness determines cellular response during MSC differentiation into osteoblasts [43,44,45]. Furthermore, the communication also works the other way around; cellular response alter ECM´s mechanical stiffness as well [46].

#### 1.4.2. MMPs as Signal Regulators

The main function of matrix metalloproteinases (MMPs), a family of zinc-dependent enzymes, is to degrade the proteins of the ECM, cleaving structural components such as collagen and gelatin. 

MMPs expression and activity are regulated at multiple levels; inactive proenzyme transcription, translation and secretion, as well as proenzyme activation or inactivation via signaling of different factors like cytokines, growth factors or even ECM proteins. Normally, secreted MMPs are synthesized as proenzymes which are activated by proteolytic cleavage of the N-terminal prodomain by serine proteases or by active MMPs. Classic activators of MMPs include the activator protein-1, nuclear factor kappa B, tumor necrosis factor- α, and the transforming growth factor beta (TGF-β) together with some interleukins. There is growing evidence showing the importance of balance amongst MMPs and their inhibitors, tissue inhibitors of metalloproteinases (TIMPs) and the membrane anchored gly coprotein RECK, for MSC fate and stage-specific expression during bone cells differentiation [47]. 

MSCs from different organs have shown differential expression and secretion of MMPs/TIMPs [48,49]. In fact, the treatment of these cells with a broad spectrum of MMP inhibitors leads to alterations in migration, proliferation, and osteoblastic differentiation, supporting that these processes are MMP dependent [48,50]. Mauney J. and Volloch V. showed that bone marrow MSCs undergoing adipogenic differentiation, express more MMPs than TIMPs [51]. However, under osteogenic differentiation conditions, cells express more TIMPs than MMPs, reinforcing the key role of MMP/TIMP balance for matrix modulation and MSC differentiation [52].

MMPs, apart from ECM degrading enzymes, have a central role regulating several signaling pathways by cleaving many circulating, cell surface and pericellular proteins irreversibly. Among the molecules that are released by MMPs, TGF-β stands out, responsible of MSCs migration to resorbed sites promoting bone formation [53]. MMP-mediated activation and release of TGF-β has been described as a negative feedback mechanism to limit MMP expression and further TGF-β release [54]. Osteoclast secretion of MMP-9 activates trapped TGF-β in the ECM, and this TGF-β may downregulate cathepsin K and MMP-9 expression; thereby controlling the amount of bone resorption that occurs by mature osteoclasts [52]. However, TGF-β can also lead to an increase in MMP-13 expression, which is related with increased osteoclast differentiation and activation [55,56]. Altogether, this evidence underlines the required tight regulation and interconnection between TGF-β and MMPs pathways to achieve a correct bone homeostasis. 

#### 1.4.3. TGF-β Signaling Pathway

As stated previously, TGF-β pathway plays a crucial role in bone metabolism regulating bone mass and quality [57]. There are more than 40 members in the TGF superfamily, including bone morphogenetic proteins (BMPs), growth and differentiation factors, activins, nodal, and Müllerian inhibitory substance [58], in addition to TGF-β1, TGF-β2 and TGF-β3 isoforms, being TGF-β1 one of the most abundant cytokines in the bone matrix [59]. 

In bone, TGF-β is produced as large precursor molecule by bone-forming osteoblasts, being composed of mature TGF-β and latency-associated protein (LAP). TGF-β remains sequestered in the ECM as an inactive, latent form since LAP remains noncovalently bound to mature TGF-β as it is secreted. However, upon osteoclastic resorption, LAP is cleaved, releasing the active TGF-β. A gradient of active TGF-β promotes the recruitment of MSCs to the recently resorbed bone surface by inducing chemotaxis and proliferation [60]. Once MSCs reach these sites, they differentiate into osteoblasts in response to environmental factors (such as bone-matrix-derived insulin-like growth factor 1) [61].

In addition to regulating the proliferation and differentiation of MSCs, active TGF-β has shown to be also an important regulator for osteoclastogenesis in a dose-dependent manner. High concentrations of active TGF-β generated at resorption areas inhibit the recruitment of osteoclast precursors, protecting it from further resorption during bone formation process [62]. Instead, low concentrations of active TGF-β induce the migration of osteoclast precursors [63]. This dual effect of TGF-β is also important in osteoclast differentiation. Low TGF-β levels stimulate osteoclast differentiation, whereas high levels inhibit such differentiation by regulating receptor activator of nuclear factor κβ ligand (RANKL)/osteoprotegerin (OPG) ratio [64]. In normal conditions, osteoblasts and osteocytes secrete RANKL, which binds to its receptor in osteoclasts (RANK) and promotes their differentiation. However, TGF-β can induce the expression of OPG in osteoblasts, a cytokine that acts as a decoy receptor for RANKL [65], thus inhibiting osteoclasts differentiation.

More recently, it has been shown that TGF-β presents both inhibitory and stimulatory effects in human osteoclast differentiation via Smad1 and Smad3 signaling, respectively [66]. These facts points out the complexity of TGF- β signaling governing the regulation of a wide range of bone metabolisms cellular functions. 

Other pivotal members of TGF superfamily are BMPs. BMPs induce MSCs differentiation into bone [67,68] via the interaction with their cell surface receptors (BMPRs) in a canonical pathway similarly to TGF-β, leading to the activation of Smads. Like TGF-β, BMPs also activate several non-Smad signaling transducers, namely, mitogen-activated protein kinase (MAPK) pathways, including extracellular signal-regulated kinases (ERKs), c-Jun amino terminal kinase (JNK), p38 MAPK, the IκB kinase, phosphatidylinositol-3 kinase and Akt, as well as Ras homolog family GTPases. 

Several studies have demonstrated that following TGF-β/BMP induction, both the Smad and p38 MAPK pathways converge at the runt-related transcription factor 2 (*Runx2*) gene to control mesenchymal precursor cell differentiation [69,70]. *Runx2* promotes the differentiation of progenitor cells into osteoblast, preventing adipogenesis [71] and exhibiting its essential role in MSC fate determination.

#### 1.4.4. Wnt Signaling Pathway

Wingless-type mouse mammary tumor virus integration site family (Wnt) is essential for skeletal formation and development, being involved in a variety of processes like differentiation, proliferation and synthesis of bone matrix by osteoblasts as well as osteoclasts differentiation and function [72,73]. In fact, alterations not only in the intensity, but amplitude, and duration of Wnt signaling pathways affects skeletal formation during development, in addition to bone remodeling, regeneration, and repair during the lifespan [74]. 

Wnts can trigger several signaling cascades, among them, the most studied is the canonical Wnt/B-catenin pathway. Briefly, Wnt elicits the stabilization and nuclear translocation of β-catenin, which is a transcription coregulator. In the absence of Wnt, β-catenin is phosphorylated by a large protein complex (adenomatous polyposis coli/Axin/glycogen synthase kinase -3β-complex), leading to its ubiquitination and proteasomal degradation through the β-TrCP/Skp pathway. However, when Wnt is secreted, it binds to membrane Frizzled receptors and triggers a cascade of several intracellular events, allowing β-catenin translocation to the nucleus, activating Wnt target genes expression [75].

Canonical Wnt signaling pathway promotes MSCs differentiation into osteoblasts by preventing apoptosis in both; osteoblast progenitor cells and differentiated osteoblast [76]. As expected, Wnt signaling is also involved in cellular lineage dichotomy; more precisely Wnt10a, Wnt10b and Wnt6 favor osteogenesis at the expense of adipogenesis; suppressing the differentiation of MSCs to adipocytes while facilitating their differentiation to osteoblasts through the canonical Wnt pathway [77,78].

As stated throughout the present review, osteoclast progenitor differentiation is tightly regulated by osteoblasts and osteocytes. In normal conditions, osteoblasts and osteocytes express RANKL, which binds to osteoclasts receptor RANK, promoting their differentiation. However, the canonical activation of Wnt signaling pathway in osteoblast-lineage cells enhances the expression of OPG, a decoy receptor of RANKL, suppressing osteoclast differentiation and thus bone resorption [79].

## 2. Bone ECM Alteration in Pathological Conditions Associated with Fragility

There are numerous diseases related to bone fragility in which ECM is altered preventing to perform its normal functions. Herein we´ll focus mainly on two bone disorders; the prevalent illness osteoporosis and the rare disease OI, both characterized by low bone mass.

### 2.1. Bone Extracellular Matrix Composition

#### 2.1.1. Bone Extracellular Matrix Composition in Osteoporosis

Osteoporosis is a worldwide disease characterized by reduction of bone mass and alteration of bone architecture [80]. According to the National Institutes of Health Consensus Development Panel on osteoporosis [81], it is defined as “a skeletal disorder characterized by compromised bone strength leading to an increased risk of fracture.” Osteoporosis mainly occurs in postmenopausal women [82] and elderly men [83], affecting approximately 200 million people [84,85,86], even more, its prevalence is expected to increase significantly in the future because of aging of the population, especially in developed nations [87]. Worldwide, approximately 8.9 million fractures are caused by osteoporotic fracture annually: over 50% of postmenopausal white women and 20% of white men will have an osteoporotic-related fracture in their lifetime [88]. Besides the health and social challenges, osteoporosis represents a major concern of the health care systems because of its growing economic burden.

The hazardous increase in the risk of fractures is a major cause of concern for the affected population [3]. Although BMD measurement is one of the most widespread diagnostic tools, the majority of fragility fractures occur in individuals whose BMD value is above the diagnostic threshold of osteoporosis [89], stressing the notion that BMD is just one among several indicators of bone health. Clearly, there is a need for improvement in the identification of patients at risk for fracture. To this extent, assessment of fracture risk should also rely on other bone properties related with bone quality [90], such as the composition of bone tissue (proportion of hydroxyapatite, water, type I collagen, and other NCPs), the biophysical properties of these components (the degree and type of collagen crosslinking, the mineral crystal size and their crystallinity), the ECM structure and the altered signaling pathways. Hence, each one of these properties may independently contribute to the increased or decreased risk of fracture, even without meaningful changes in BMD.

As an example of inorganic matrix bone composition of implication, it has been shown that the degree of trabecular bone matrix mineralization is slightly reduced in premenopausal women with idiopathic osteoporosis (osteoporosis of under 50 years adults with unknown cause) compared to normal controls [91]. Concerning organic matrix, *COL1A1* gene is implicated in reduced BMD in osteoporosis; in fact, type I collagen polymorphisms (Sp1 [92] and +1245G/T [93]) play a role in development of osteoporosis and fracture. Thus, osteoporosis may alter the collagen alignment and mineral geometry in bone formed before and after the onset of this disease [94]. 

NCPs regulate the matrix assembly and play a significant role in the structural organization of bone, thus influencing its mechanical properties [95]. NCPs levels vary during aging and disease such as osteoporosis, leading to an increased fracture risk. That way, alteration in NCPs such OPN, OC and ON could increase the risk of developing osteoporosis [96]. The role of OPN in bone remodeling has been confirmed by studies on osteoporosis development in which ovariectomized (OVX) OPN^+/+^ mice lost bone mass, while OVX OPN^−/−^ mice showed higher bone volume than the earlier. Furthermore, when OVX mice were treated with anti-OPN antibody, a marked inhibition in bone loss was observed, along with a reduction in the number of resorptive areas [97]. These animal studies are in agreement with observed in affected patients, in which higher levels of serum OPN were found in postmenopausal women with osteoporosis, compared to nonaffected ones [98,99]. 

OC is considered a marker of bone formation, therefore several epidemiological studies tried to establish the role of serum and urinary OC detection as an accurate biomarker for osteoporosis. However, the results are at least controversial. In spite some studies have reported that osteoporotic women have increased serum OC [100,101,102], recently data revealed no significant difference in serum OC level between postmenopausal osteoporosis cases and controls [39]. This could be explained by OC molecules are quite heterogeneous in the circulation and can be influenced by glucose metabolism. 

The third NCPs that is considered, ON has an important role in matrix regulation and mineralization, making ON a good candidate for the osteoporosis onset [103]. In fact, ON-null mice have decreased bone formation in addition to decreased osteoblast and osteoclast surface (the proportion of the bone covered with osteoblasts or osteoclasts) and number, leading to decreased bone remodeling with a negative bone balance, causing profound osteopenia [104]. Moreover, ON polymorphisms seem to affect BMD in humans. Delany and coworkers observed that the haplotype commonly associated to a high bone density is mainly expressed in normal subjects than in osteoporosis patients, while the expression of ON haplotype associated to low BMD is higher in osteoporosis patients than in controls [105]. 

Finally, both collagen [106] and NCPs undergo different post-translational modifications, which alter the quality of the ECM and the response of bone to mechanical load. Hence, bone matrix protein phosphorylation levels are tightly related with bone fracture risk. To address this gap, a recent study has demonstrated that as people age, the total phosphorylation level declines by approximately 20% for bone matrix proteins [107]. Moreover, these outcomes suggest that the decline of total protein phosphorylation of ECM contributes to bone fragility and could lead to development of osteoporosis. 

#### 2.1.2. Bone Extracellular Matrix Composition in Osteogenesis Imperfecta

Osteogenesis imperfecta (OI), also called “brittle bone disease”, is a rare genetic disorder characterized by an increased susceptibility to bone fractures and decreased bone density [88]. In the majority of cases, it is caused by mutations in the *COL1A1* or *COL1A2* genes and, as expected, associated to abnormality in the synthesis and/or processing of type I collagen. Nowadays, mutations in up to 19 different genes have been identified in a dominant and recessive traits [108,109]. Besides the genetic heterogeneity, OI presents a wide clinical variability [110] in where clinical manifestations range from mild, with a nearly asymptomatic form, to most severe one resulting in perinatal mortality [111]. Genetic mutations that cause a quantitative reduction of type I collagen cause milder forms of OI disease, and conversely, structural mutations of type I collagen, significantly affect the quality of the bone matrix resulting in moderate to lethal forms of OI. 

Since type I collagen is the major component of bone being essential for bone mineralization, it can be assumed that changes in collagen quantity or quality will have detrimental effects on mineralization. In line with this observation, several studies have claimed that OI patients have higher average mineralization densities than age-matched healthy controls [112,113], and increased BMD distribution [114]. It has been suggested that the higher mineralization is a consequence of abnormal OI ECM assembly, which results in increased water fraction that is available for mineral deposition. Thus, although mineralization in OI patients is increased, bones are brittle due to an alteration in bone ECM formation and structure.

Moreover, there are OI patients presenting mutations in specific genes that directly affect the bone mineralization process such as *IFITM5* (Interferon induced Transmembrane Protein 5) [115] and *SERPINF1* (Serpin Family F Member 1) [116]. Histological studies on iliac crest biopsy specimens from these OI patients described lamellae with irregular organization and a meshlike appearance [117,118]. Although the defective proteins that encode the genes mentioned are not involved in the synthesis of type I collagen there is evidence for reduced type I collagen production and increased mineralization in primary osteoblast cultures [119], even hypermineralization of bone tissue [120]. 

Regarding the organic matrix, OI patients present a reduced quantity of bioactive type I collagen due to alterations in multiple processes that contribute to its synthesis, secretion, processing, assembly and interaction with other matrix components, as mentioned previously [121]. As type I collagen is the most abundant protein in bone ECM, its normal level reduction will affect ECM composition and in consequence, ECM functions. 

On the other hand, several alterations in NCP levels have been reported in OI patients [122,123]. First, bone sialoprotein, OC [124] and alpha 2-HS glycoprotein concentrations were found increased in cortical bone from OI patients [125]. OI cell matrices present not only a reduced level of proteins such as collagen, but also a decrease in decorin [126] and ON [127]. Moreover, mutations in *SPARC* (the gene encoding ON) have been identified in individuals with recessive OI [128], which demonstrated severe bone fragility, pointing out that the collagen-binding function of ON plays a critical role in collagen deposition in bone [129]. 

Osteoblasts from OI cultures exhibit reduced amounts of insoluble collagen deposition and increased synthesis of the glycosaminoglycan hyaluronan when compared to ECM deposited by osteoblasts from control individuals [130]. Defects in collagen secretion or deposition that might contribute to the fragility of the OI bone by interfering with complete mineralization and/or normal tissue architecture [129].

All in all, OI affects the ECM in multiple ways [121] and the study of OI has clearly highlighted the essential role of material properties in bone strength [131].

### 2.2. Bone Extracellular Matrix Structure

#### 2.2.1. Bone Morphology in Osteoporosis

Osteoporosis animal models and human patients share not only bone mass reduction but alteration of bone architecture. Cortical bone of the mid-diaphysis in OVX mice (murine model of postmenopausal osteoporosis), show a reduced tissue mineral density and increased cortical porosity, this later feature also exhibited by OVX rat model of postmenopausal osteoporosis rat (Sprague-Dawley). An increase in cortical vascular porosity may diminish bone strength as well as alter bone mechanotransduction via interstitial fluid flow, both of which could contribute to bone fragility during postmenopausal osteoporosis [132]. These results are in agreement with osteoporosis patients outcomes in which the cortical bone becomes more and more porous with increasing age, and therefore, the largest loss of absolute bone mass is in cortical bone [29]. Since cortical bone plays a major role in determining the mechanical competence of bone and the risk of fracture, the age related alterations of its geometrical features and its local porosity may alter bone strength and lead to bone brittleness.

Trabecular bone has been also shown to be affected in osteoporosis. The number of trabeculae, the trabecular thickness and the degree of connectivity influence the mechanical strength of a bone. Thus, in OVX mice (murine model of osteoporosis), the trabecular bone volume of the distal-metaphysis is decreased [133]. Moreover, in male Wistar rats subjected to orchiectomy bone loss is observed too. Separately, the orchiectomy led to significant tomographic alterations in the trabecular bone number, trabecular separation, and trabecular pattern factor [134]. The same results have been reported in early osteoporosis patients, since the bone loss is mainly a trabecular deficiency, and a decrease of all these characteristics is observed [29]. Bones with increased risk for osteoporotic fractures, present remaining trabecular tissue largely heterogeneous [135], with regions of different mineralization, stiffness and strength. For example, it was demonstrated that bone structure deterioration of the tibial plateau due to osteoporosis was region-specific [136], and the greatest decrease in bone volume fraction was found in the medio-medial segments and the lowest bone volume was found in central segments (tibial spine). It has been proposed that these changes are a transient and site-specific characteristic of osteoporosis, whereby the trabecular tissue properties are altered varyingly as the disease progresses. In addition, changes in the distribution of mechanical stimuli related to changes in the microarchitecture of the trabecular have been studied [137]. Variations in the morphology of the trabecular bone have been predicted, such as an increase of 30% in porosity, which significantly altered the distribution of mechanical stimuli of the environment where the cells are located. These results suggests that changes in the microarchitecture cause a proportional decrease in the mechanical stimuli that may drastically affect the mechanoregulation of bone regeneration, promote microcracks and accelerate osteoporosis. 

#### 2.2.2. Bone Morphology in Osteogenesis Imperfecta

Bone morphology has also been studied in patients suffering from OI [138], presenting BMD, trabecular volumetric BMD, total bone area, and cortical bone area lower in OI than in healthy age- and gender-matched controls [139]. Higher tissue mineral density was found for OI bone, a dramatically rise in cortical porosity, canal diameter, and connectivity [140] along with a lower elasticity [141]. That way, compared to control group, the cortical thickness seems to be thinner in OI patients [142]. 

With respect to trabecular microstructure in patients with OI, significantly lower trabecular parameters including bone volume fraction (BV/TV) and bone trabecular number (Tb.N) [142], as well as increased trabecular spacing were observed in comparison to the control group. A tendency toward thicker trabeculae was found [139]. These observed results are possibly due to increased bone turnover that in turn increases trabecular perforations and thus leads to preferential loss of thin trabeculae [143]. Taking everything into account, the mutation-induced collagen defects alter the collagen matrix, thereby affecting the mineralization and leading to increased brittleness. 

### 2.3. Bone Extracellular Matrix Biophysical Properties

#### 2.3.1. Bone Extracellular Matrix Biophysical Properties in Osteoporosis

Though osteoporosis is generally defined as a loss of bone mass, there are considerable matrix changes, particularly in collagen crosslinks, which cause a loss of bone quality [3,18]. Enzymatic crosslinks have been shown to be reduced in osteoporotic patients with hip fractures compared to healthy controls [144,145]. Moreover, collagen from the femoral head of osteoporotic women has a higher degree of hydroxylated lysine residues (formed from nonenzymatic crosslinks) than that from nonosteoporotic women [146]. 

Intermediate product generated from the nonenzymatic crosslink named AGEs accumulate with age and disease [17], so it is not unexpected that osteoporotic bone presents significantly more AGEs than normal healthy bone [144,147,148]. The activation of the RAGE (AGE receptor) inhibits osteoblast proliferation and differentiation [149], reduces matrix production [150], reduces bone formation [151] and increases osteoblast apoptosis [152], finally deteriorating bone’s mechanical properties. All things considered, crosslinking properties of the matrix may alter the tissue properties and therefore play an important role in the decreased bone formation found in osteoporosis [153].

How menopause affects bone quality has been and is an intense research line, given the tight link between menopause and the development of osteoporosis. Thus, an analysis of bone matrix quality from healthy women with and without menopause pointed out that women with menopause demonstrate a decrease in mineral/organic ratio, microhardness, mineral maturity and crystallinity, suggesting the alteration of local mineral content and microhardness [154], in spite that the mean degree of mineralization was no different. Outcomes that are in line with previous study, in which microhardness was significantly lower in osteoporotic patients compared with controls [155]. 

#### 2.3.2. Bone Extracellular Matrix Biophysical Properties in Osteogenesis Imperfecta

When the secreted collagen type I has altered post-translational modification, leading to defective crosslinking [156], the collagen reduces its ability to correctly bind to other matrix molecules. Mutations in genes that are involved in collagen crosslinking have been reported in OI patients [157], such as *SERPINH1, FKBP10* and *PLOD2* [123]. 

*SERPINH1* encodes heat shock protein 47 (Hsp47), a collagen-specific molecular chaperone. Hsp47 transiently associates with triple-helical procollagens in the endoplasmic reticulum (ER) and dissociates at the cis-Golgi, returning to the ER via its ER retention signal [158]. Mutations in Hsp47 and consequent impairment of the chaperone function in the ER, lead to overhydroxylation and partial intracellular retention of procollagen I. Both consequences, ER stress and aberrant bone collagen crosslinking, underlie the OI pathology associated to crosslinking defects, but further studies are required [159]. 

Bruck syndrome is a disorder characterized by joint flexion contractures and skeletal dysplasia that shows strong clinical overlap with OI but is caused by biallelic mutations in either the *FKBP10* or the *PLOD2* genes [160,161,162]. *PLOD2* encodes lysyl hydroxylase 2 (LH2), the enzyme responsible for hydroxylation of collagen telopeptide lysine. Telopeptide hydroxylysines are essential for the hydroxyallysine pathway of crosslinking, which produces mature crosslinks in extracellular collagen fibrils, crucial for the normal material properties of bone [163]. *FKPB10* encodes FKBP65, an ER resident peptidylprolyl isomerase that functions as a molecular chaperone that aids in the folding of type I procollagen [164]. Moreover, it is involved in collagen crosslinking by specifically mediating the dimerization of LH2, which is required for its activity [158,165]. Type I collagen isolated from *FKBP10 knockout* mice revealed less stable crosslinks [166]. In the absence of *FKBP10*, collagen fibrils deposited in matrix are sparse and disorganized, consistent with the defect in crosslinking [167]. Thus, collagen monomers not able to crosslink may simply dissociate from fibers due to a low collagen concentration in media, leading to bone fragility and deformity. Overall, mutations in genes related with collagen processing and consequent impaired collagen crosslinking, lead to bone fragility, as can observe in some bone brittle patients. 

In order to delve into the understanding of the high bone matrix mineralization observed in patients with OI, mineral composition (mineral particles size, alignment and mineral-to-matrix ratio) were analyzed. Studies in the *oim* murine model (OI mouse) demonstrated that the hydroxyapatite crystals are thinner and less-well aligned along collagen fibrils compared to controls [168]. That way, *oim* bones have lower stiffness that may result from the poorly organized mineral matrix with significantly smaller, highly packed and disoriented apatite crystals [169]. The same results were reported in OI patients: the size of mineral particles was the same or smaller than controls [170], but their packing density was increased [171]. Moreover, Raman spectroscopy showed that the mineral-to-matrix ratio was higher in OI samples, while the crystallinity was lower [141], suggesting that the mineral crystals were smaller but more abundant in the case of OI. These changes in crystal size, distribution and composition contribute to the observed decrease in mechanical strength and consequent bone fragility. 

To sum up, studies on OI bones have shown increased bone mineralization, accompanied by hydroxyapatite crystals that are reduced in size, more densely packed and less-well organized along collagen fibrils. These features lead to changes in intrinsic bone material properties with the consequent increase in bone brittleness [131].

The following table summarized the different ECM alteration at matrix composition, microarchitecture and biophysical properties level in osteoporosis and OI (Table 1).

### 2.4. Altered Bone Extracellular Matrix Signaling in Bone Pathologies

#### 2.4.1. Integrins

The integrity of collagen type I molecule has revealed to be essential for a correct binding to integrin cell receptors. Not all subunits that form integrins are equally affected in osteoporotic patients, for instance, α2 integrin, which is part of α2β1 heterodimer, is downregulated, in osteoporotic patients [172]. 

Another central aspect for this molecular interaction is the collagen triple helical conformation. This structure requires a glycine as every third residue to stabilize it, generating the characteristic (Gly-Xaa-Yaa)*n* sequence [173]. The glycine at that position is essential, since even a single Gly substitution by another residue leads to OI phenotype [174]. Hamaia and colleagues, elucidated the precise (Gly-Xaa-Yaa)*n* amino acid sequence in collagen required for integrin binding [175]. An interesting study focused on α2β1 integrin and how Gly mutations affect their integrin-collagen binding [174], pointed out that mutations occurring in crucial regions of collagen binding motifs avoid integrin interaction and, consequently, cell adhesion, leading to severe or lethal OI phenotypes.

#### 2.4.2. MMPs 

Osteoclast activity is associated with an increase in MMP-9 expression which stimulates osteoclasts resorption and degrades ECM matrix proteins like collagen type I [176]. MMP-9 serum levels have been found to be overexpressed in osteoporotic bones [177]. In addition, high levels of MMP-2, as well as fragments derived from bone collagen by the cleavage of MMP-2, have been found in the circulation of osteoporotic patients [178]. Thus, in osteoporosis patients, the excessive MMPs activity and therefore osteoclasts activity is targeted by bisphosphonates, which reduce bone resorption by inhibiting the enzymatic activity of MMPs in osteoclasts. Some examples are the bisphosphonates based on highly selective MMP-2 inhibitors [179,180] and a TIMP-2-based MMP-14 inhibitor [181].

On the other hand, BMP1 is a metalloproteinase known to have procollagen C-proteinase activity that cleaves the C-propeptides from procollagens I-III [182]. Mutations of the *BMP1* gene lead to defects in collagen processing and are associated with OI in humans [183,184,185] and other animal species such as mouse and zebrafish [183,186,187], emphasizing the importance of all proteins involved in collagen processing to achieve a correct bone formation. Studies of mesenchymal stem cells have demonstrated that the collagen-binding α2β1 and α11β1 integrins both have an impact on osteogenic differentiation [172].

#### 2.4.3. TGF-β

**Figure 1:**
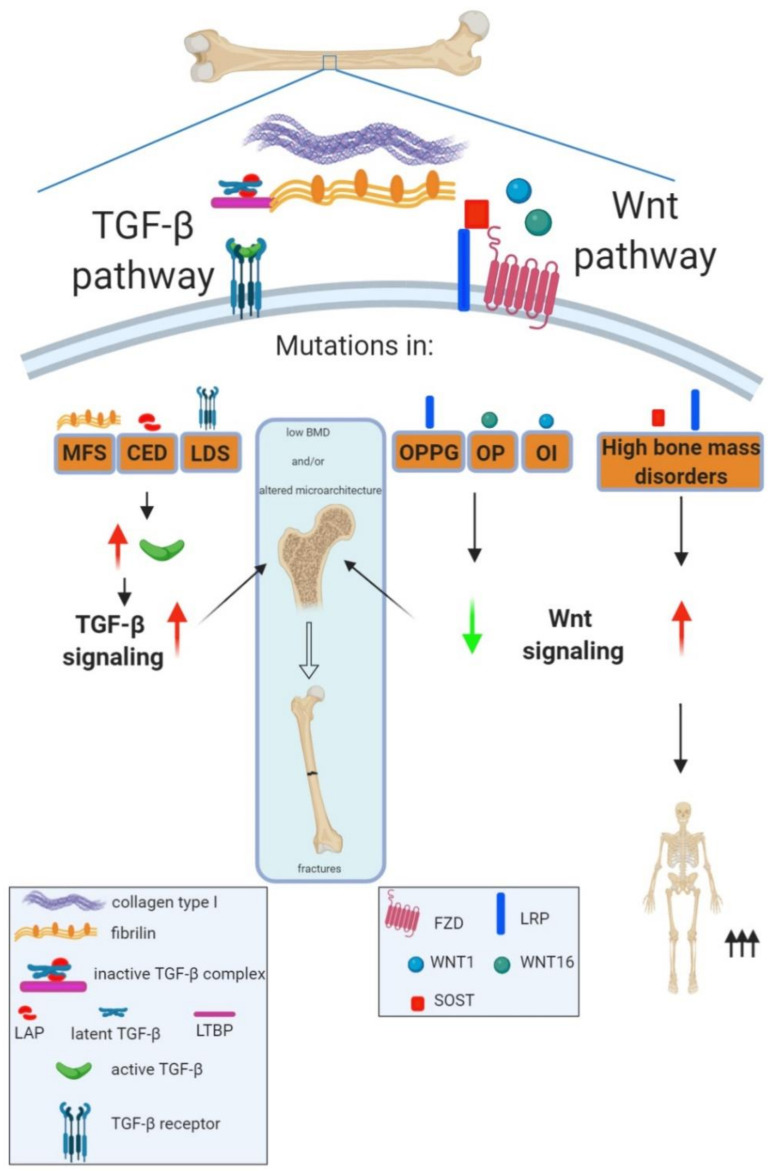
Roles of transforming growth factor beta (TGF-β) and Wnt signaling in bone strength maintenance. Bone strength depends on bone mineral density and bone microarchitecture; disturbances in any of them lead to increased risk of fractures. Human musculoskeletal disorders with mutations in TGF-β or Wnt family members have revealed the key role of these pathways in regulating bone strength. Thus, mutations in different TGF-β family members leading to an increased pool of active TGF-β and therefore an increased TGF-β signaling give rise to rare disorders characterized by low bone mineral density, alterations in bone microarchitecture and increased risk of fractures. This is the case of Marfan (MFS) and Loeys–Dietz (LDS) syndromes and Camurati–Engelmann disease (CED), with mutations in *FBN*1 (Fibrillin-1), TGFβ receptors (*TGFβR1* and *TGFβR2*) and *LAP*, respectively. In the case of the Wnt pathway, mutations leading to increased Wnt signaling can cause high bone mass disorders, whereas inactivating mutations of the Wnt pathway are associated to low bone mass disorders with different ranges of severities. Thus, heterozygous mutations in Wnt1 or SNPs in Wnt16 can lead to osteoporosis (OP) whereas homozygous mutations of Wnt1 cause osteogenesis imperfecta (OI). Moreover, autosomal recessive loss of function mutations in LRP5 are known to cause the rare osteoporosis pseudoglioma syndrome (OPPG) characterized by extremely severe childhood onset osteoporosis. For definitions of other abbreviations, please see the main text. Red arrow increased expression; green arrow decreased expression.

The existence of a fine-tuned bone ECM signaling to maintain bone microstructure becomes unquestionable given the existence of a number of bone pathologies with alterations in key signaling pathways, as an underlying pathological mechanism. Of relevance is the case of the TGF-β signaling pathway, previously remarked, which is modulated by bone ECM proteins. A number of mutations have been identified in different members of the signaling pathway, leading to an array of rare genetic diseases, all of them sharing skeletal alterations such as compromised trabecular and cortical bone microarchitecture (Figure 1).

Marfan syndrome (MFS), a connective tissue disorder with a wide range of musculoskeletal and cardiovascular alterations, is caused mainly by mutations in *FBN1* gene, which codes for Fibrillin-1, an ECM structural protein which polymerizes into microfibrils. Fibrillin-1, which in bone tissue represents less than 3% of ECM proteins, binds to latent TFG-β-binding proteins forming large latent TGF-β complexes. Mutations in *FBN1* gene lead to an increased pool of active TGF-β and therefore to an enhanced TGF-β signaling, featuring lower bone mass exhibited by affected individuals [188,189]. However, a high clinical variability is observed in MFS patients, and, in fact, more than 1800 different mutations in *FBN1* (only 12% recurrent) have been described [190]. Recently, alterations in microarchitectural parameters have also been described in long bones from MFS adults: trabecular bone shows reduced trabecular number and thickness as well as higher trabecular separation, whereas cortical bone shows reduced thickness and increased porosity [191]. It is still unclear whether there is an increased risk of fractures in MFS patients, recent studies point to an increased fracture risk in both pediatric and adult MFS patients [172,192,193]. The increased fracture rate was not found in pediatric patients with the lower mineral density, suggesting that an altered bone microarchitecture could be playing a pivotal role in MFS fracture risk [192]. On the contrary, a recent study, found that, in spite of the bone microarchitecture alterations, the estimated bone strength in MFS was similar to that expected for healthy controls. This in silico prediction could be explained by the fact that MFS bones exhibit an increased longitudinal growth, which could be counteracting the consequences of having deficiencies in bone mass and microarchitecture [191]. 

Loeys–Dietz syndrome (LDS) is caused mainly by mutations in genes coding for TGF-β receptors, *TGF-βR1* and *TGF-βR2*. Mutations are predicted and/or in vitro verified to inhibit the kinase activity of TGF-β receptors and therefore diminish the activation of the pathway, but paradoxically aortic tissues of LDS patients show elevated TGF-β signaling [194]. In fact, patients show clinical features that overlap with those shown by MFS patients, suggesting a common pathogenic mechanism, although in this case, patients show more aggressive vascular disease. As regards skeletal tissue, patients show thinner cortical bones, low BMD and increased risk of fractures [195,196] but, up to now, there are no studies addressing bone microarchitecture in these patients. Mouse models carrying mutations in *TGF-βR2* recapitulate the low bone mass phenotype of LDS patients, resembling severe human LDS [197]. Cortical bone at the femoral shaft of mice proved to be especially affected, with decreased bone area and cortical thickness, whereas trabecular bone of the distal femur showed no differences compared with controls. The greater affectation of cortical bone could explain the major risk of fractures in long bones, pointing to a key role of TGF-β pathway in cortical bone maintenance.

Camurati–Engelman disease (CED), a rare skeletal dysplasia, is caused by mutations in the N-terminal prodomain (LAP) of *TGF-*β*1*, leading to an increase TGF-β signaling [198]. The cortical thickening of the diaphysis of long bones of the upper and lower limbs is the hallmark of the disease. The existence of increase fractures in CED patients is controversial and there are no studies regarding their microarchitecture in which could estimate bone strength. However, a transgenic mouse model of CED exhibiting cortical thickness increased as in humans, showed a higher incidence of long bone fractures along with increased cortical bone porosity [53].

These rare pathologies affecting bone show that overexpression of TGF-β signaling pathway deeply affects bone microstructure leading to an increased risk of fractures. Interestingly, cortical bone tissue seems to be especially affected in these pathologies characterized by TGF-β signaling hyperactivation, emphasizing TGF-β’s crucial role in maintaining cortical bone tissue homeostasis.

In OI, one study that used two distinct mouse models of OI showed that excessive TGF-β signaling is an important disease mechanism that contributes to the OI phenotype [199]. Although the precise process that leads to TGF-β overactivity has not been worked out yet, it is likely that type I collagen and consequent proteoglycan alterations in OI cause inefficient retention of TGF-β in the matrix. 

Regarding osteoporosis, several TGF-β superfamily members (*TGFB1*, *BMP2*, *BMP4*, and *Sclerostin*) [200] have been implicated as candidate genes in osteoporosis [201]. Indeed, several polymorphisms in *TGF-β1*, related with higher serum TGF-β1 levels and significantly lower bone mass, have been identified in patients with osteoporosis [202]. In addition, another study identified single nucleotide polymorphisms (SNPs) in several TGF-β pathway components in patients with osteoporosis (*TGFBR1*, *TGFBR2*, *Smad2*, *Smad3*, *Smad4*, and *Smad7*) [203], but the relevance of these polymorphisms remains to be established.

#### 2.4.4. Wnt

The bone-anabolic role of Wnt pathway was revealed by the existence of high bone mass disorders along with the identification of causative gene mutations in key Wnt family members, responsible for Wnt signaling cascade hyperactivation. This was the case of patients with heterozygous gain of function mutations in lipoprotein receptor-related protein 5 (*LRP5*) (a coreceptor of the Wnt pathway [204]), or loss-of-function mutations of its inhibitor sclerostin [205]. 

Conversely, loss-of-function mutations in LRP5 cause autosomal recessive osteoporosis-pseudoglioma syndrome (OPPG) characterized by blindness from birth, very low bone mass in early childhood and consequent increased risk of fractures and bone deformation [128]. 

In this regard, mutations in the fourth β-propeller of *LRP5* have been associated with early childhood-onset primary osteoporosis [206,207] (Figure 1). Interestingly, lithium, a known Wnt pathway activator (mentioned above) is being currently in a clinical trial for OPPG (ClinicalTrials.gov Identifier: NCT01108068).

Later studies identified that mutations or even variants in the sequence of Wnt ligands can lead to osteoporosis with different ranges of severities [208]. Thus, a genomewide association study (GWAS) identified a specific SNP of *Wnt16*, which was associated to individuals with decreased cortical bone thickness and forearm BMD as well as increased fractures [209]. In the same way, heterozygous Wnt inactivating mutations can lead to low bone mass and early onset osteoporosis [210,211,212]. For instance, rare variations in the *Wnt3a* and *DKK1* genes have been observed in patients with childhood-onset primary osteoporosis [213]. Indeed, Wnt3a-mediated signaling stimulates bone formation and inhibits bone resorption in OVX mice [214], pointing out Wnt3a as a potential therapeutic target for osteoporosis treatment. In particular, Wnt pathway has been reported as a target in osteoporotic treatments [215], the best therapeutic targets for promoting Wnt signaling are the several secreted endogenous inhibitors that constantly exert a negative influence on this pathway to keep it tamed [216]. Thus, by targeting these inhibitors one can activate Wnt signaling and increase bone mass [217]. Inhibition of sclerostin, a Wnt antagonist secreted by osteocytes, leads to decreased bone resorption and increased bone formation. Monoclonal antibodies against sclerostin (romosozumab [218,219]) have proven in clinical trials to be a very efficient osteo-anabolic approach to the treatment of osteoporosis, since they increase bone formation, bone mass, and bone strength [220], therefore decreasing markedly fracture risk in treated patients. Another Wnt ligand related with bone microarchitecture is Wnt1, since mutations in the *Wnt1* gene shows reduced trabecular and cortical parameters especially cortical thickness. Interestingly, complete loss of function of Wnt1, due to homozygous or compound heterozygous mutations, leads to moderate-to-severe OI [221]. 

## 3. Conclusions

In conclusion, bone ECM is tightly related to bone strength, and alterations of different ECM factors could lead to diseases associated with bone fragility. Indeed, the skeleton derives its resistance to fracture from multiple components regulated across several levels of hierarchical organization. 

Firstly, the different cells and molecules that compose bone matrix affect bone resistance and, therefore, are implicated in bone fragility. Thus, alterations in bone composition may affect bone mechanical properties and thereby fracture risk. For instance, alterations in type I collagen molecules give rise to OI, a rare disease characterized by bone fragility. 

Bone microarchitecture has a crucial role in determining bone resistance. Cortical bone is densely packed, providing the strength and rigidity to bones, whereas trabecular bone is less mineralized, organized to optimize load transfer and disperse the energy of loading. That way, different cortical (tissue mineral density and porosity) and trabecular (the number of trabeculae, trabecular thickness and heterogeneity) parameters are altered in osteoporosis and OI, leading to a more brittle bone. 

Then, biophysical properties of the bone ECM, such as the degree and type of collagen crosslinking, the mineral crystal size and their crystallinity, are important determinants of cell behavior. Cells respond differently to denatured collagen than mature, crosslinked collagen fibrils. In osteoporosis, bone quality is affected, since there are considerable changes in collagen crosslinks, which cause loss of bone quality. In OI, the secreted collagen type I can have altered post-translational modification, leading to defective crosslinking. This collagen reduces its ability to correctly bind to other matrix molecules, leading to an altered collagen network that is not able to support mechanical stimuli. Regarding mineral crystal size and alignment, hydroxyapatite crystals are thinner and less well-aligned along collagen fibrils in OI patients compared to controls, leading to decreased mechanical strength and consequent bone fragility.

Finally, ECM components provide a complex network of biochemical and physiological signals to bone cells that contribute to bone homeostasis. In this way, TGFβ and Wnt signaling pathways have been found to be major contributors to bone strength. Thus, the identification of different bone disorders in which basal signaling of these pathways is altered, has been of crucial relevance in order to decipher the role of TGFβ and Wnt pathways in bone strength.

All in all, the relative composition, organization and maturity of the mineral and organic bone matrix, together with the cell-matrix signaling, determine the correct development, maintenance and functionality of bone tissue.

## Figures and Tables

**Table 1:** Alterations in extracellular matrix (ECM) composition, structure and biophysical properties in osteoporosis and osteogenesis imperfecta (OI).

				Osteoporosis	OI
Composition	Inorganic matrix	Mineralization		↓	↑
		Mutated genes		N/A	*IFITM5, SERPINF1*
	Organic matrix	Type I collagen		*COL1A1* *	↓
		NCPs	OPN	OPN ^+/+^ → bone mass ↓ OPN ^−/−^ → bone volume ↑	N/A
			OC	↓	↓
			ON	ON ^−/−^ → bone formation ↓ ON * → BMD ↓	*↓* Mutations in *SPARC*
Structure	Cortical	TMD		↓	↑
		Porosity		↑	↑
		Elasticity		N/A	↓
		Areal BMD, cortical bone area		N/A	↓
	Trabecular	Bone volume		↓	↓
		Bone trabecular number		↓	↓
		Porosity		↑	N/A
		Trabecular spacing		N/A	↑
		Tissue heterogeneity		↑	N/A
		Thickness		N/A	↑
Biophysical properties	Collagen crosslinking	Enzymatic		↓	N/A
		Nonenzymatic		↑	N/A
		Mutated genes		N/A	*SERPINH1, FKBP10, PLOD2*
	Mineral composition	Mineral/organic ratio		↓	N/A
		Mineral/matrix ratio		N/A	↑
		Microhardness		↓	N/A
		Mineral particles	Size	N/A	=/↓
			Packing density	N/A	↑
		Crystallinity		↓	↓

* Polymorphisms; ↓ decrease; ↑ increase; ^+/+^ wild type; ^−/−^ knockout, =/↓ similar or decrease; N/A, not available; for definitions of other abbreviations, please see the main text.

## Abbreviations

advanced glycation end-products	AGEs
bone mineral density	BMD
bone morphogenetic proteins	BMPs
Camurati–Engelman Disease	CED
endoplasmic reticulum	ER
extracellular matrix	ECM
heat shock protein 47	Hsp47
latency-associated protein	LAP
lipoprotein receptor-related protein 5	LRP 5
Loeys–Dietz syndrome	LDS
lysyl hydroxylase 2	LH2
Marfan Syndrome	MFS
matrix metalloproteinases	MMPs
mitogen-activated protein kinase	MAPK
noncollagenous proteins	NCPs
osteocalcin	OC
osteogenesis imperfecta	OI
osteonectin	ON
osteopontin	OPN
osteoporosis-pseudoglioma syndrome	OPPG
osteoprotegerin	OPG
ovariectomized	OVX
parathyroid hormone	PTH
receptor activator of nuclear factor κβ ligand	RANKL
runt-related transcription factor 2	Runx2
single nucleotide polimorfism	SNP
transforming growth factor beta	TGF-β
tissue inhibitors of metalloproteinases	TIMPs
wingless-type mouse mammary tumor virus integration site family	Wnt
